# Inferring Gene Regulatory Networks Using Conditional Regulation Pattern to Guide Candidate Genes

**DOI:** 10.1371/journal.pone.0154953

**Published:** 2016-05-12

**Authors:** Fei Xiao, Lin Gao, Yusen Ye, Yuxuan Hu, Ruijie He

**Affiliations:** School of Computer Science and Technology, Xidian University, Xi'an, Shaanxi 710071, China; National Institute of Genomic Medicine, MEXICO

## Abstract

Combining path consistency (PC) algorithms with conditional mutual information (CMI) are widely used in reconstruction of gene regulatory networks. CMI has many advantages over Pearson correlation coefficient in measuring non-linear dependence to infer gene regulatory networks. It can also discriminate the direct regulations from indirect ones. However, it is still a challenge to select the conditional genes in an optimal way, which affects the performance and computation complexity of the PC algorithm. In this study, we develop a novel conditional mutual information-based algorithm, namely RPNI (Regulation Pattern based Network Inference), to infer gene regulatory networks. For conditional gene selection, we define the *co-regulation pattern*, *indirect-regulation pattern* and *mixture-regulation pattern* as three candidate patterns to guide the selection of candidate genes. To demonstrate the potential of our algorithm, we apply it to gene expression data from DREAM challenge. Experimental results show that RPNI outperforms existing conditional mutual information-based methods in both accuracy and time complexity for different sizes of gene samples. Furthermore, the robustness of our algorithm is demonstrated by noisy interference analysis using different types of noise.

## Introduction

Inferring gene regulatory networks is a key step in understanding biological processes [1–5]. Microarray techniques generate a large amount of gene expression data, providing a workable data foundation [6]. Many computational methods were developed to infer gene regulatory networks using these high-throughput data [2, 4]. These methods can be divided into two categories: the model-based and the machine learning-based approaches [3].

Model-methods are based mainly on singular value decomposition [7], multiple linear regression [8] and linear programming [9]. In machine learning methods, Bayesian networks, Pearson correlation coefficient, partial correlation coefficients, information theory, and conditional mutual information are applied to measure the regulation strength between genes. Bayesian networks are based on maximizing the scoring function, for the moment, dynamic programming is the best way to achieve a global optimal structure with 35 nodes [10]. Although Cassio *et al*. [11] proposed a structure constraint method based on Bayesian information criterion (BIC) and Akaike information criterion (AIC), reducing the size limitation to 70 nodes, it remains an open problem due to its local optimum and high computing cost [3, 12, 13]. Pearson correlation coefficient and information theory can reconstruct large-scale networks with limited samples in acceptable time [14, 15]. Compared with Pearson correlation coefficient, mutual information (MI) provides a reasonable gauge to measure non-linear dependence (which commonly exists in biology [16]). Therefore, mutual information is widely applied in inferring gene networks [3, 16–20].

In recent years, conditional mutual information (CMI) has taken the place of MI because MI cannot distinguish the direct interactions from the indirect ones [17–19, 21]. Path consistency (PC) algorithms are an effective strategy to infer a causal network by conditional relation [14, 18, 19, 22]. Combining PC algorithm with CMI and corrected-CMI, PCA-CMI (path consistency algorithm based on conditional mutual information) [18] and CMI2NI (CMI2-based network inference) [17] are proposed to “thin” the edges with independent correlation recursively from zero to high order correlation. Theoretical analysis shows that CMI underestimates the regulatory strength in some cases [23]. CMI2 corrects the underestimation by utilizing interventional probability and KL-divergence (Kullback—Leibler divergence), however, previous methods force to select conditional genes which has exponential complexity w.r.t the data size, so it is still a challenge to select the conditional genes in an optimal way [18], which may affect the performance and sharply reduce the search space [22].

In this work, we aim to define three candidate patterns based on biological processes [24, 25] to guide the selection of candidate genes. A novel algorithm, called RPNI (Regulation Pattern based Network Inference), is developed to infer gene regulatory networks by considering the candidate patterns and PC algorithm based on CMI2 to delete the edges with independent correlation recursively. We also make statistical analysis using different scales of yeast networks. Z-tests show that our defined candidate patterns significantly exist in gene regulatory networks, consistent with the discovered regulation motifs [23, 24]. Our method also greatly reduces the computational complexity. Under the hypothesis of Gaussian distribution of gene expression data, CMI2 can be calculated in a simple form using a covariance matrix of related gene expression data [18]. RPNI follows CMI2’s strength to measure the regulatory strength. Moreover, it can accurately predict regulatory networks using limited samples. We apply our algorithm to DREAM data [2, 26, 27], and experimental results show that RPNI outperforms PCA-CMI and CMI2NI in both accuracy and time complexity. Furthermore, the robustness of our algorithm is demonstrated by noisy interference analysis using different types of noise.

## Methods

This section includes an introduction to some definitions of information theory, a path consistency algorithm, our defined candidate patterns and the RPNI algorithm for inferring gene regulatory networks.

### Information theory

With the advantages of measuring non-linear dependence association between two variables and relatively high efficiency, information theory is increasingly used to measure the regulatory strength between genes. The definitions of mutual information (MI) and conditional mutual information (CMI) are as follows:

$$MI(X,Y)=\iint p(x,y)\text{log}\frac{p(x,y)}{p(x)p(y)}dxdy$$ (1)

$$CMI(X,Y|Z)=∭p(x,y,z)\text{log}\frac{p(x,y|z)}{p(x|z)p(y|z)}$$ (2)

where *p*(*x*,*y*) denotes the joint distribution of *X* and *Y*. *p*(*x*) and *p*(*y*) represent the marginal distribution of *x* and *y*, respectively. Since it is widely accepted that gene expression data follow Gaussian distribution [18, 19], formulation of entropy subject to n-dim Gaussian distribution can be easily calculated by a simple equation, where |*C*| is the determinant of covariance matrix of variables *x*_1_,*x*_2_,…,*x*_*n*_ [28].

$$H(X)=\text{log}{\left(2\pi e\right)}^{\frac{n}{2}}{\left|C\right|}^{-\frac{1}{2}}$$ (3)

After mathematical transformation, we can obtain the following equation, guiding us to compute MI and CMI2.

$$MI(X,Y)=\frac{1}{2}\text{log}\frac{\left|C(X)\right|\times \left|C(X)\right|}{\left|C(X,Y)\right|}$$ (4)

CMI2 proposed to integrate Kullback—Leibler divergence [28] and interventional probability in order to correct the underestimation of CMI [23],

$$CMI2(X,Y|Z)=\sum_{x,y,z}p(x,y,z)\text{ln}\frac{p(x,y,z)}{p(x,z)\sum_{x}p(y|x,z)p(x)+p(y,z)\sum_{y}p(x|z,y)p(y)}$$ (5)

With the same hypothesis of Gaussian distribution, CMI2 can be easily calculated. The details of computational process and mathematical proof can be found in Zhang’s work [18].

### Path consistency algorithms

Path consistency (PC) algorithms are widely used in inferring gene regulatory networks [14, 18, 19]. By removing the most likely uncorrelated edges repeatedly from low to high order dependence correlation until it can’t continue, PC-algorithm can construct a high-confidence undirected network [22].

### Candidate Pattern

We define the *co-regulation pattern*, *indirect-regulation pattern* and *mix-regulation pattern* to facilitate the selection of candidate genes in inferring gene regulatory networks.

**Fig 1:**
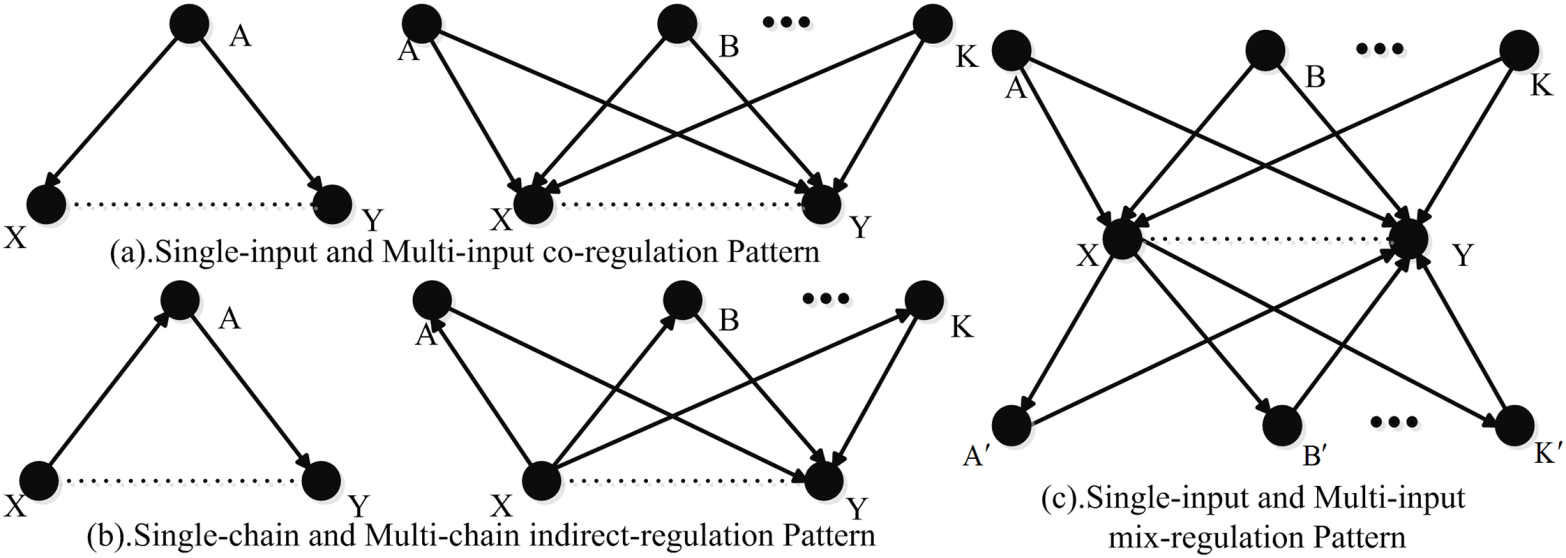
Diagram of candidate patterns. (**a**) *Co-regulation pattern* consists of single-input and multi-input co-regulation. (**b**) *Indirect-regulation pattern* consists of single-chain and multi-chain indirect-regulation. (**c**) *Mix-regulation pattern* includes both co-regulation and indirect-regulation.

Single-input co-regulation pattern (also denoted as the single input motif) is defined as a pattern in which a set of target genes are regulated by a single gene (Fig 1a), in other words, two or more genes share the same upstream gene in this pattern and guide the deleting of false positive (FP) edges [18]. Single-input co-regulation pattern occurs infrequently in randomized networks (*p<*0.01) and is potentially useful for coordinating a discrete unit of biological function. For example, several genes in the leucine biosynthetic pathway are regulated by the Leu3 transcriptional regulator [23]. In Fig 1a, gene *X* and gene *Y* have a common upstream gene *A* (i.e. gene *A* regulates gene *X* and gene *Y* at the same time). This causes gene *X* and *Y* to have higher mutual information (MI), which leads to false positives. Choosing *A* as the conditional gene can significantly reduce the MI between *X* and *Y* and guide the deleting of false positive (*FP*) edges [19]. As an extension of co-regulation, we take into account the situation of more than one regulator, whose structure is denoted as the *multi-input co-regulation pattern* [24]. Experiment indicates that the sets of genes regulated by different transcription factors in *E*. coli share much more common genes than expected at random [25] for both cases. In this scenario, their co-regulators are selected as conditional genes. Both single-input co-regulation pattern and multi-input co-regulation pattern are collectively called co-regulation pattern.

Second, *single-chain and multi-chain indirect-regulation pattern* is defined as a pattern in which a gene is both directly or indirectly regulated by two or more genes. As is shown in Fig 1b, target gene *Y* is both directly and indirectly regulated by gene *X* and gene *A*. This structure is also denoted as the *regulator chain motif*. It consists of a chain in which one regulator binds the promoter of a second regulator and the second binds the promoter of a third regulator, and so forth. This network motif is observed frequently in the location data for yeast regulators [25]. As mentioned above, MI cannot distinguish the direct interactions or correlations from indirect ones, which leads to FP [19]. Here, choosing gene *A* as the candidate gene can guide the deleting of FP edges. Both single-chain and multi-chain indirect-regulation pattern are collectively called indirect-regulation pattern.

Third, in *the mix-regulation pattern* (Fig 1c), gene *X* and gene *Y* are affected by both co-regulation and indirect-regulation. It is evident that choosing genes in {*A*,*B*,…,*K*} and {*A'*,*B'*,…,*K'*} as the conditional genes can remove the *FP* edges between gene *X* and gene *Y*. The case containing co-regulation pattern and indirect-regulation pattern simultaneously is called mix-regulation pattern.

### RPNI

**Fig 2:**
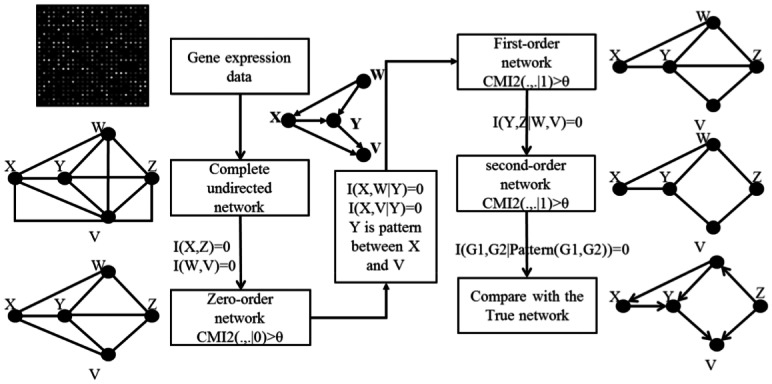
Diagram of RPNI. First, we generate a complete undirected graph. Second, we choose independent edges by MI between any two nodes. If MI is smaller than *θ*, the corresponding edge will be deleted. Here, *I*(*X*,*Z*) and *I*(*W*,*V*) are equal to zero, so the edges *E*(*X*,*Z*) and *E*(*W*,*V*) are deleted and we obtain the zero—order network. The first-order network is then constructed by deleting *E*(*X*,*V*) because *I*(*X*,*V|Y*) = 0 and *Y*,*X*,*V* satisfy the *co-regulation pattern*. Based on *n*-order network, we construct the *n*+1-order network by deleting the conditional uncorrelated edges with the evidence of *I*(*A*,*B*|choosing any *n*+1 combination in pattern (*A*,*B*)). The algorithm terminates after construction of the second-order network because there are no enough regulation pattern genes to compute the third-order conditional mutual information.

Given an expression dataset with n genes and m samples, we develop a novel algorithm, called RPIN, to infer gene regulatory network. Firstly, we focus on the identification of the three patterns. Under the hypothesis of Gaussian distribution, for a perturbed gene, we use z-tests to select differentially expressed genes as its upstream genes. As shown in Fig 1a, in the *co-regulation pattern*, gene A is the co-upstream gene of gene X and Y, in the *indirect-regulation pattern*, gene A is the upstream gene of gene X and downstream gene of gene Y. We thus select gene A is candidate gene of gene X and gene Y. Fig 2 illustrates the flow chart of our RPNI algorithm, using a network consisting of five genes as an example. First, we generate a complete undirected graph with five nodes. Second, we choose independent edges by MI between any two nodes. If MI is smaller than *θ*, the corresponding edge will be deleted. Here, *I*(*X*,*Z*) and *I*(*W*,*V*) are equal to zero, so the edges *E*(*X*,*Z*) and *E*(*W*,*V*) are deleted and we obtain the zero—order network. The first-order network is then constructed by deleting *E*(*X*,*V*) because *I*(*X*,*V*|*Y*) = 0 and *Y*,*X*,*V* satisfy the *co-regulation pattern*. Based on *n*-th order network, we construct the *n*+1-th order network by deleting the conditional uncorrelated edges with the evidence of *I* (*A*,*B*|choosing any *n*+1 combination in pattern (*A*,*B*)). The algorithm terminates after construction of the second-order network because there are not enough regulation pattern genes to compute the third-order conditional mutual information. The detail procedure of this algorithm is described in Box 1.

**Box 1**

**Algorithm RPNI**

**Input**:

Gene expression matrix *A*,

Dependence threshold *θ*.

**Output**:

Inferred gene regulatory network *G*,

Regulatory strength of each edge,

Order of inferred network *L*.

**Step-1**. Initialization. Generate a complete connected network *G*_0_. Set *L*: = −1.

**Step-2**. Choose candidate gene set. *L*: = *L+1*; For each existing edge (i.e. *G*_0_(*i*,*j*)≠0), select adjacent gene connected with both gene *i* and *j*. If their common neighbors form a pattern, add it to candidate gene set. Compute the number of genes (*T*) in the candidate gene set.

**Step-3**. Set *G*: = *G*_0_. If *T*<*L*, stop the algorithm; else, select L genes from these *T* genes and add each combination into the set $K=\left\{k_{1},k_{2},\ldots ,k_{n}\right\},n=C_{T}^{L}$, compute these *CMI*2(*i*, *j*|*k*) and choose the maximal one denoted as *CMI*2_max_(*i*, *j*|*k*). If *CMI*2_max_(*i*, *j*|*k*)<*θ*, set *G*(*i*,*j*) = 0.

**Step-4**. If *G* = *G*_0_, stop the algorithm; else, set *G*_0_ = *G* and return to Step-2.

## Results

### Datasets and evaluation metrics

In order to compare our method with CMI and CMI2, we apply these methods to infer gene regulatory networks using the same dataset from DREAM3 challenge and acute myeloid leukemia (AML) based on the Level-3 processed RNA sequencing data of AML patient from TCGA (http://cancergenome.nih.gov/) [29, 30].

The performance of the methods is evaluated using true positive rate (*TPR*), false positive rate (*FPR*), positive predictive value (*PPV*), accuracy (*ACC*) and Matthews coefficient constant (*MCC*) [18]. Their definitions are as follows:

$$TPR=\frac{TP}{\left(TP+FN\right)}$$ (6)

$$FPR=\frac{FP}{\left(FP+TN\right)}$$ (7)

$$PPV=\frac{TP}{\left(TP+FP\right)}$$ (8)

$$ACC=\frac{\left(TP+TN\right)}{\left(TP+FP+TN+FN\right)}$$ (9)

$$MCC=\frac{\left(TP\times TN-FP\times FN\right)}{\sqrt{\left(TP+FP)(TP+FN)(TN+FP)(TN+FN\right)}}$$ (10)

where *TP*,*FP*,*TN* and *FN* denote the number of true positives, false positives, true negatives and false negatives, respectively.

We also plot the receiver operating characteristic (ROC) curves and calculate the area under curve (AUC) [18, 31] which is the area under the ROC. Finally, we compare the running time between our method and CMI2 in the same parameter and environment.

### Performance on simulation data

**Fig 3:**
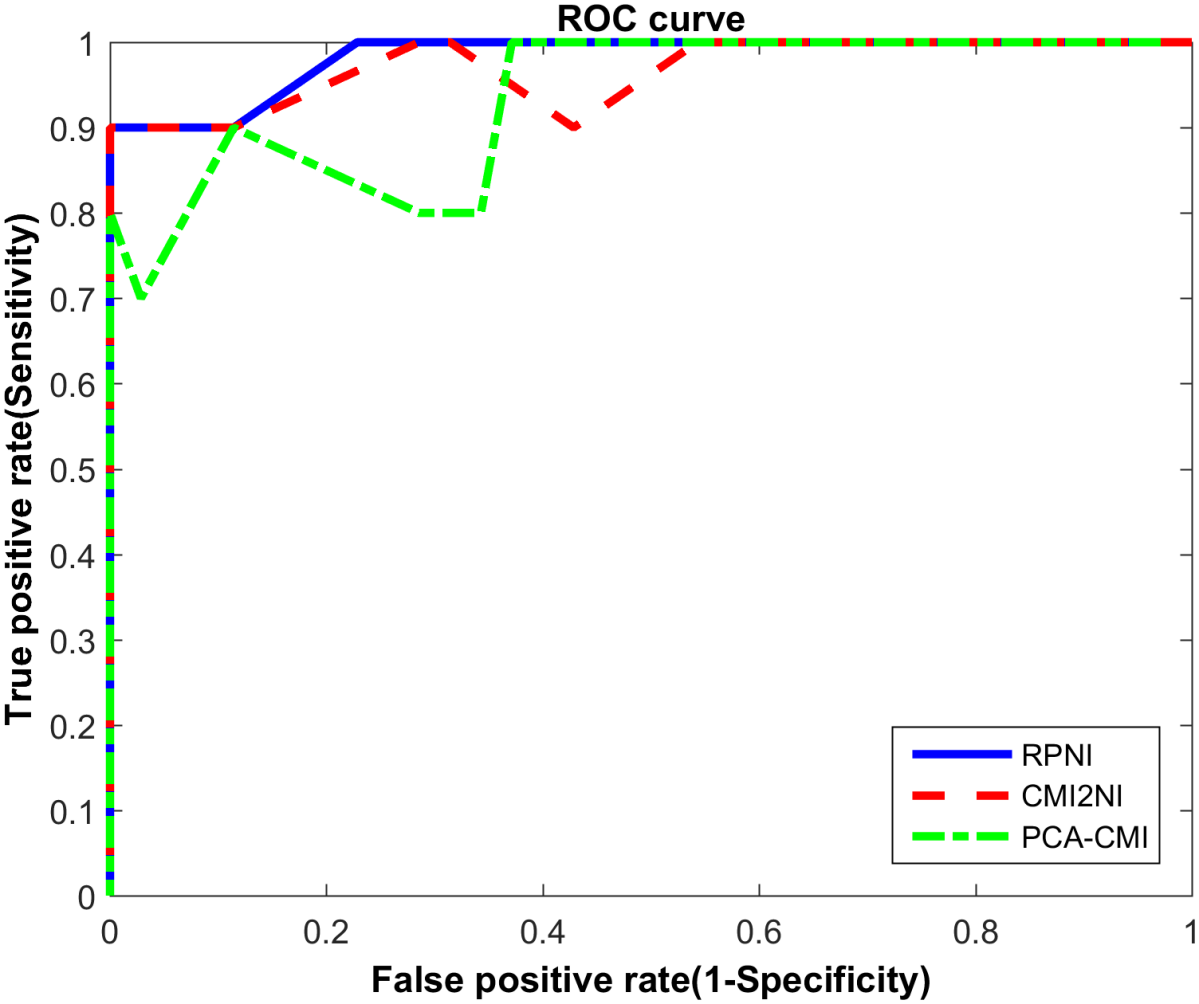
ROC curves generated using several methods including RPNI, CMI2NI, PCA-CMI on DREAM3 challenge Yeast1 dataset in size 10. The blue solid line is the ROC curve of RPNI. The AUC value reaches 0.9929.

We use the gene expression data from DREAM3 challenge, which aims at reconstruction of gene networks from steady state data. There are three sub-challenges corresponding to three gene networks with 10 and 50 genes. To validate the performance of our method, we apply it to different sizes of networks and compare the performance between different methods. We choose the null-mutants data which contain the steady state levels for the wild-type and the null-mutant strains for each gene. We test RPNI on Yeast1 gene expression data with 10 genes and 11 samples, and choose 0.03 as the threshold to delete edges. The detailed results are shown in Table 1, and the ROC curves are plotted in Fig 3, which shows that RPNI is superior to both PCA-CMI and CMI2NI.

**Table 1:** Comparison of different methods using networks with sizes 10, 50.

Method	TP	FP	TN	FN	TPR	FPR	PPV	ACC	MCC	AUC
**Size 10**										
PCA-CMI	9	1	34	1	0.9	0.028	0.9	0.956	0.8714	0.9343
CMI2NI	9	1	34	1	0.9	0.028	0.9	0.956	0.8714	0.9757
RPNI	9	1	34	1	0.9	0.028	0.9	0.956	0.8714	**0.9929**
**Size 50**										
PCA-CMI	29	34	1114	48	0.377	0.029	0.46	0.933	0.3813	
CMI2NI	32	31	1117	45	0.416	0.027	0.508	0.938	0.427	
RPNI	42	43	1105	35	0.545	0.037	0.494	0.936	**0.4852**	

Second, we test RPNI on Yeast1 gene expression data with 50 genes and 51samples. We choose the same threshold of 0.05 as in Zhang’s work [17] to delete edges. The detailed results are listed in Table 1.

**Fig 4:**
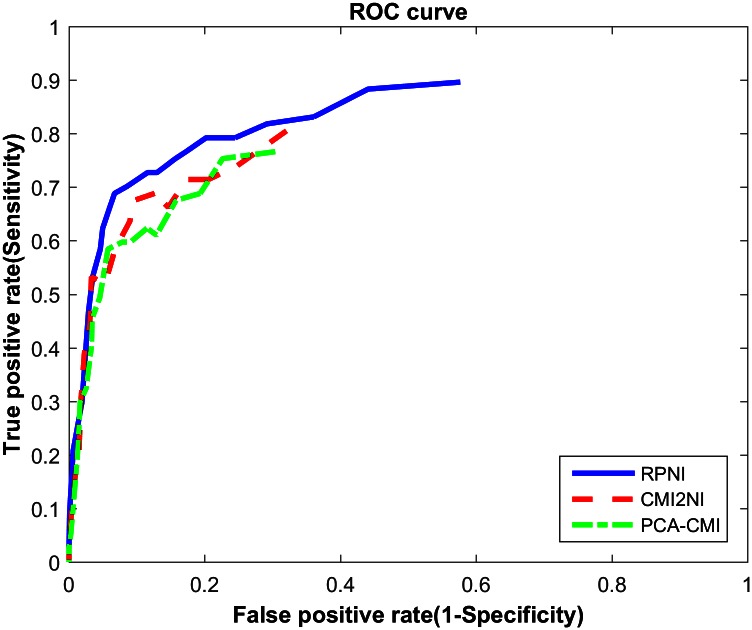
ROC curves of several methods on DREAM3 challenge Yeast1 dataset in size 50.

The time complexity of our algorithm for a graph G is bounded by the largest degree in *G*. Let *k* be the maximal degree of any vertex and let *n* be the number of vertices. Then, in the worst case, the *T*(*n*) of CMI2 required by the algorithm is bounded by $\frac{n^{2}{\left(n-1\right)}^{k-1}}{(k-1)!}$. The closer to 0 the threshold, the closer to $\frac{n^{2}{\left(n-1\right)}^{k-1}}{(k-1)!}$ the calculation counts [22]. So we cannot compare the complete ROC curves because when the threshold is too small, the time complexity will reach $O\left(\frac{50^{2}{\left(49\right)}^{47}}{(47)!}*t\right)$. Assuming one second can accomplish 100000 counts of CMI2 (actually it is 1000 times per second on Intel i5-3470 3.20GHz). The computation time of this algorithm is 8.4E10 years. Based on the above discussion, we conclude that it is meaningless when threshold is too small, leading to deleting few edges. Fig 4 shows the ROC curve with the parameter ranging from 0.001 to infinity. ROC curve shows our method outperforms other methods [19] in accuracy.

### Case study: cancer regulatory networks

As a case study, we construct a gene regulatory network for cancer which can provide a global view of disease-causing gene regulations [32]. We thus use RPNI to build a gene regulatory network for acute myeloid leukemia (AML) based on the Level-3 processed RNA sequencing data of AML patient from TCGA (http://cancergenome.nih.gov/) [29, 30]. The RPKM value is used as the gene expression level.

**Fig 5:**
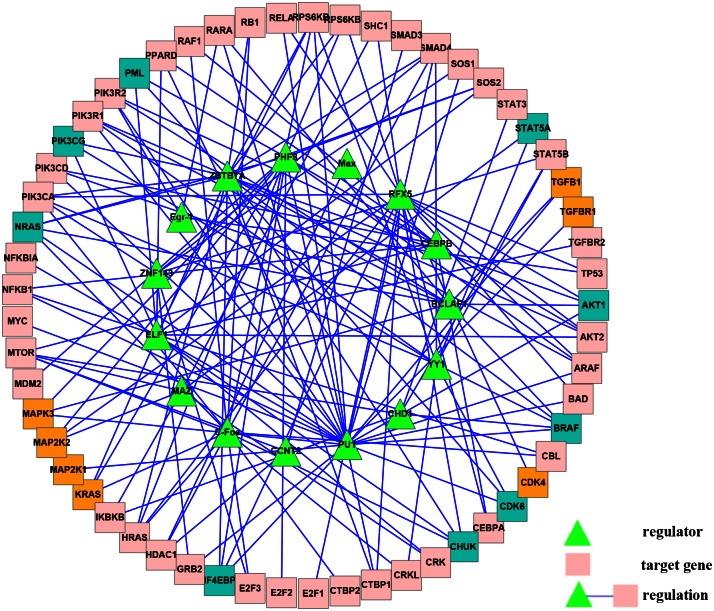
AML-specific gene regulatory network reconstructed by CMI2NI and RPNI. The common target genes are colored in pink. The differential target genes inferred by RPNI and CMI2NI are colored in blue and yellow, respectively.

We constructed an AML-specific regulatory network with RPNI considering the 81 cancer genes involved in a network built by RACER [33]. Our reconstructed network consists of 16 regulators, 65 target genes, and 151 regulatory links, showed in Fig 5, among which 33 regulatory links have also been inferred by RACER [32].

In order to show the superiority of RPNI, the same gene expression data are used as input for CMI2NI to infer a regulatory network. The comparative results show that our algorithm generates 16 differential regulation links compared with CMI2NI and that the network generated using CMI2NI includes 14 differential regulation relationships. In order to verify the effectiveness of these differential regulating relationships, we hereby verify how many genes in these two target genes sets are involved in the pathways related to the AML. By analyzing the two differential target gene sets using cancer gene annotation system CaGe (http://mgrc.kribb.re.kr/cage/), we noticed that 8 in 9 target genes with RPNI and 4 in 7 target genes with CMI2NI are related to the AML. Apparently, the target gene set inferred by RPNI is more significantly enriched for AML cancer pathways than that inferred by CMI2NI. Our method has a significantly better p-value (p-value = 2.1742e-16) than CMI2NI’s p-value (p-value = 1.9473e-07), statistical test using the method of fisher exact test (the detailed results are listed in Table 2).

**Table 2:** Enrichment analysis results using RPNI algorithm.

No.	Base Pathways^a)^	Pathway (Database)^b)^	Genes in pathway	Genes overlapped	p-value^c)^	q-value^d)^
1	ALL	ACUTE MYELOID LEUKEMIA (KEGG)	60	8	2.04E-16	1.70E-13
2	ALL	CHRONIC MYELOID LEUKEMIA (KEGG)	73	7	4.31E-13	1.79E-10
3	ALL	PATHWAYS IN CANCER (KEGG)	328	8	1.73E-10	4.80E-08
4	ALL	ERBB SIGNALING PATHWAY (KEGG)	87	6	2.95E-10	6.14E-08
5	ALL	NON SMALL CELL LUNG CANCER (KEGG)	54	5	3.24E-09	5.40E-07
6	ALL	GLIOMA (KEGG)	65	5	8.35E-09	1.16E-06
7	ALL	PANCREATIC CANCER (KEGG)	70	5	1.22E-08	1.36E-06
8	ALL	MELANOMA (KEGG)	71	5	1.31E-08	1.36E-06
9	ALL	PROSTATE CANCER (KEGG)	89	5	4.08E-08	3.77E-06
10	ALL	INSULIN SIGNALING PATHWAY (KEGG)	137	5	3.46E-07	2.60E-05

^a)^ Pathway set used for the test. CGC: 146 Cancer Gene Censers gene-based pathways, CGI: 179 Cancer Gene

^b)^ Index gene-based pathways, and ALL: All 833 pathways from BioCarta/KEGG/Reactome databases.

^c)^ p-value from Fisher's exact test for the overlapping gene.

^d)^ q-value for false discovery rate control.

### Robustness study

**Fig 6:**
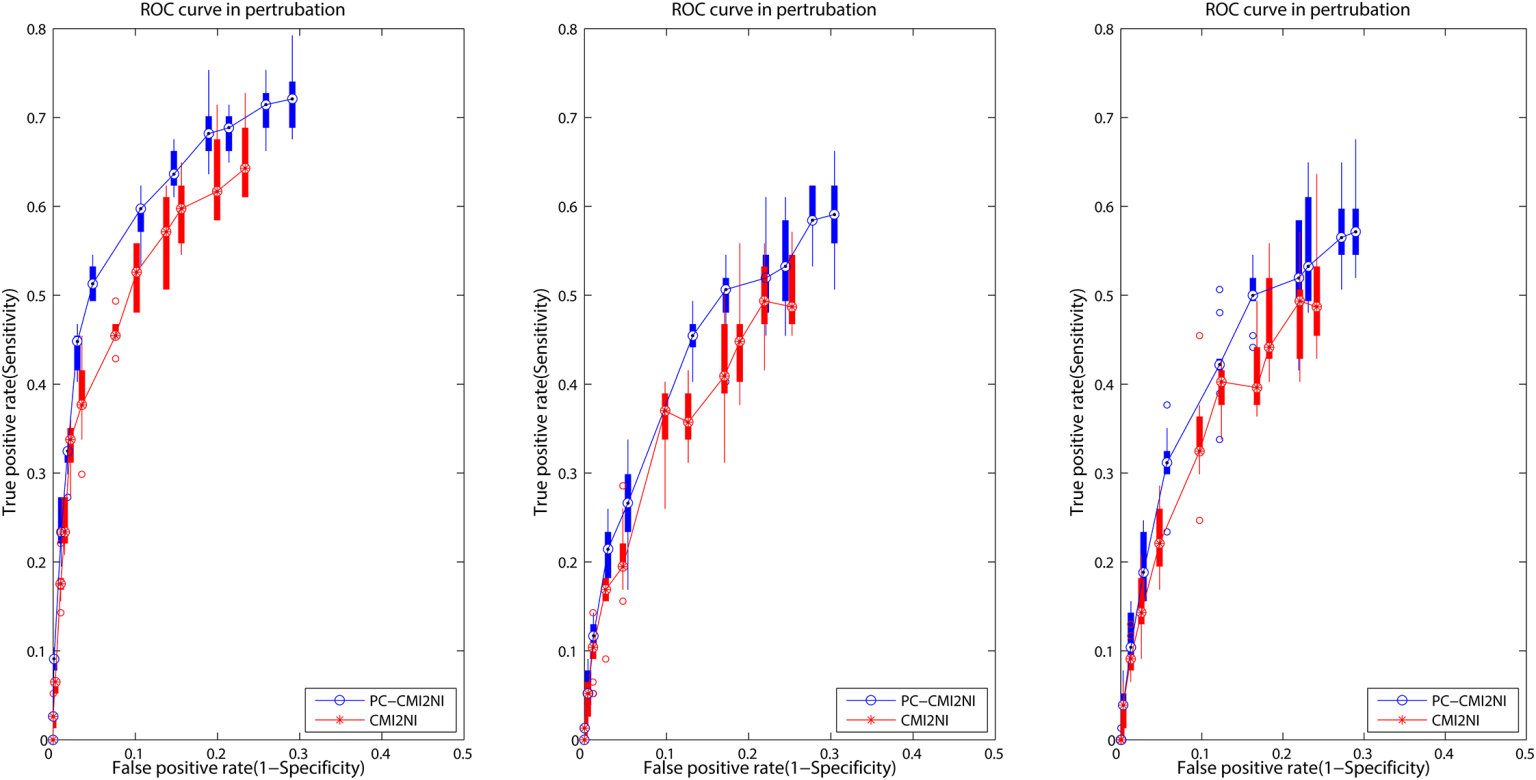
ROC curves of two methods of CMI2NI and RPNI using different types of noise. (A) ROC curves of two methods with noise. (B) ROC curves of two methods with outliers. (C) ROC curves of two methods with perturbation.

We use different types of noise to demonstrate the robustness of our algorithm. First, we use measurement errors, which follows the Gaussian distribution. Considering that different genes follow different Gaussian distribution, we add a noise of Gaussian distribution to the k-th gene whose mean is 0 and variance is $\frac{\sigma (k)}{2}$ (*σ*(*k*) is the *k*-th gene’s variance). For each parameter, we repeat this procedure ten times and compute the mean of *FPR* and the median of *TPR* as the label of *x* and *y*, respectively. For showing the result more comprehensive, we add the box and whisker chart for each point to indicate the *TPR* range for each *FPR*. Fig 6a shows that our approach outperforms CMI2NI in robustness. Moreover, we find that our method has smaller variance in *TPR*, demonstrating its good robustness in noise. Second, outlier noise is also considered here, which often leads to recording errors or instrument errors. We replace one-tenth original expression data with noise data following Gaussian distribution with the mean and variance of all expression data. As analyzed above, we plot the box and whisker chart in the ROC curve in Fig 6b. Simulation result shows that the performance of both methods are significantly decreased, nevertheless, our result is still ahead of CMI2NI in this case. Finally, we conduct a perturbation analysis. We choose one-tenth expression data and perturb their positions randomly. This procedure is also repeated 10 times. The ROC curve reveals our method is superior to CMI2NI using perturbation data.

## Discussion

Information theory-based methods show a strong ability to measure non-linear dependence that exists commonly in biology. PC algorithm is an effective strategy to “thin” the inferred graph by removing edges from zero order to higher order conditional independent relations. Due to these advantages, PCA-CMI [18] and CMI2NI [17], combining PC algorithm with CMI and CMI2, show a good performance. However, both CMI and CMI2 have not yet solved the challenge of how to select the conditional genes in an optimal way. In this paper, we propose three candidate patterns, namely *co-regulation pattern*, *indirect-regulation pattern* and *mix-regulation pattern*, to guide the choice of candidate genes. Choosing reasonable conditional genes may improve the performance of PC algorithm. Actually, not limiting candidate genes will lead to deleting some true positive edges for random noise, which is a key barrier in improving the accuracy of regulatory network inference. On the basis of CMI2, we propose a novel network inference algorithm, namely RPNI, to infer gene regulatory networks. Selecting candidate gene set sharply reduces the search space in PC algorithm simultaneously. Experimental results show that RPNI outperforms the state-of-art approaches in both accuracy and time complexity.

Despite the advantages of RPNI, there exist several promising directions to further improve its performance. First, RPNI cannot infer the direction of edges in the network. Combining Bayesian network model with RPNI may overcome this weakness. Second, choosing a biological significance pattern will improve the precision of inferred regulatory networks.

## Supporting Information

S1 File The sample data set used in the paper. (TSV)

S2 File The benchmark for the sample data set. (TXT)
